# SGLT2 inhibitor use and disparities in all-cause mortality in type 2 diabetes: insights from a multi-ethnic population

**DOI:** 10.1007/s00125-026-06733-2

**Published:** 2026-04-27

**Authors:** Lynne Chepulis, Han Gan, David Simmons, Mark Rodrigues, Rawiri Keenan, Rinki Murphy, Tim Kenealy, Leanne Te Karu, Dianna Magliano, Jo Scott-Jones, Allan Moffitt, Chunhuan Lao, Ross Lawrenson, Ryan G. Paul

**Affiliations:** 1 Medical Research Centre, University of Waikato, Hamilton, New Zealand; 2 School of Computing and Mathematical Sciences, University of Waikato, Hamilton, New Zealand; 3 School of Medicine, Western Sydney University, Sydney, NSW Australia; 4 Faculty of Medical and Health Sciences, University of Auckland, Auckland, New Zealand; 5 Health New Zealand / Te Whatu Ora Te Toka Tumai, Auckland, New Zealand; 6 School of Public Health and Preventative Medicine, Monash University, Melbourne, VIC Australia; 7 Midlands Health Network, Hamilton, New Zealand; 8 Procare Health Limited, Auckland, New Zealand; 9 Health New Zealand / Te Whatu Ora Waikato, Hamilton, New Zealand

**Keywords:** Diabetes complications, Empagliflozin, Ethnicity, Inequality, Māori, Mortality, Pacific, SGLT2 inhibitor, SGLT2i medication survival rate, Type 2 diabetes

## Abstract

**Aims/hypothesis:**

Sodium-glucose cotransporter 2 inhibitors (SGLT2i) are known to reduce cardiovascular and all-cause mortality in people with type 2 diabetes, but there are limited data regarding mortality outcomes in different ethnic groups (including Indigenous peoples). This study reports on mortality outcomes in a population in Aotearoa New Zealand (hereafter New Zealand) with type 2 diabetes, following the funded availability of the SGLT2i empagliflozin with prioritised access for Māori and Pacific people.

**Methods:**

Data were collected from primary care records for those aged 18–75 years with type 2 diabetes (Auckland/Waikato regions of New Zealand; February 2021 to December 2023; *n*=59,505). These data were linked to national medication-dispensing and mortality records for 2021–2024 via national health identifier numbers. Following propensity matching and Cox modelling for ethnicity, age, gender, medication use, baseline HbA_1c_ and cardiovascular and/or renal disease/risk (CVRD) status (yes/no), mortality rates were compared by ethnicity in those with and without CVRD and who did/did not initiate empagliflozin. This study was reported in accordance with the CONSIDER statement, used to strengthen the reporting of research involving Indigenous peoples. The study was funded by the Health Research Council of New Zealand.

**Results:**

Following matching, two groups of 12,792 individuals were identified. Annualised crude mortality (deaths per 1000 individuals per year) was higher in those not dispensed with SGLT2i than in those receiving SGLT2i (35.2 vs 13.1 in those with CVRD and 7.7 vs 3.6 in those without CVRD, respectively). After adjustment, the greatest difference in mortality with SGLT2i use was seen in Māori (HR 0.475; 95% CI 0.336, 0.672; *p*<0.001), followed by Pacific people (HR 0.507; 95% CI 0.395, 0.651; *p*<0.001) and European people (HR 0.667; 95% CI 0.545, 0.816; *p*<0.001).

**Conclusions/interpretation:**

The protective effect of SGLT2i use on mortality appears to differ by ethnicity and is greater in Indigenous Māori and Pacific populations in New Zealand with type 2 diabetes. SGLT2i use in Indigenous and minority populations may support improved health equity.

**Graphical Abstract:**

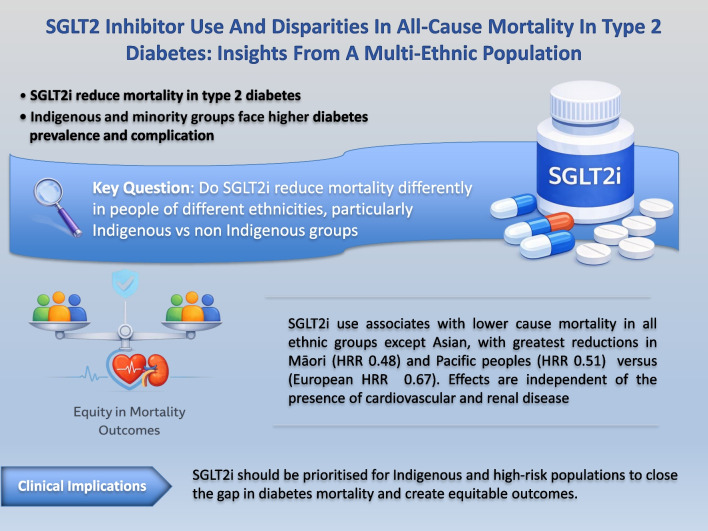

**Supplementary Information:**

The online version contains peer-reviewed but unedited supplementary material available at 10.1007/s00125-026-06733-2.


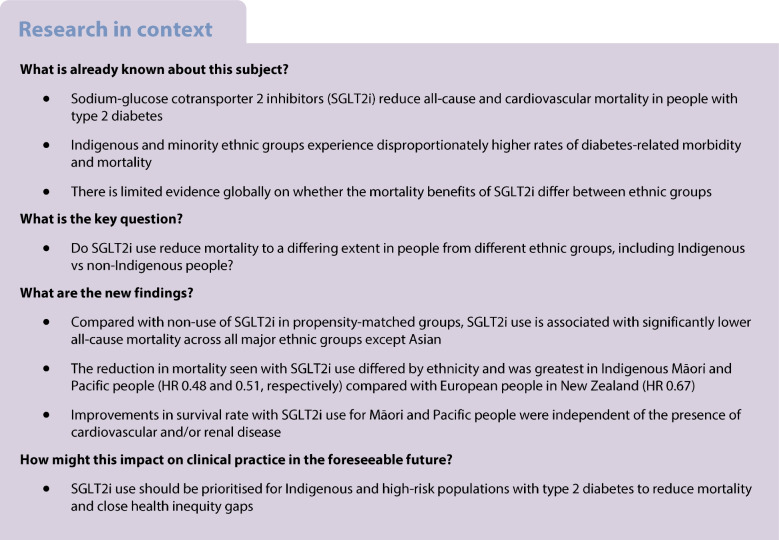


## Introduction

Sodium-glucose cotransporter 2 inhibitors (SGLT2i) have emerged as a transformative class of medications in the management of type 2 diabetes mellitus, offering benefits that extend beyond glycaemic control [1]. In particular, SGLT2i have demonstrated significant cardiovascular and renal benefits [1]. Large-scale clinical trials have shown, for example, that SGLT2i use can reduce the risk of cardiovascular events in individuals with type 2 diabetes and established cardiovascular disease (CVD) [2, 3]. This is significant, as there is an elevated risk of both all-cause and cardiovascular-related mortality associated with type 2 diabetes [4, 5], and CVD is reported to be the leading cause of death among individuals with type 2 diabetes [6, 7]. However, studies suggest that the SGLT2i empagliflozin has been shown to reduce the risk of cardiovascular death, hospitalisation for heart failure and the slow progression of nephropathy in individuals with type 2 diabetes and established cardiovascular and/or renal disease/risk (CVRD) [8].

In Aotearoa New Zealand (hereafter New Zealand), the prevalence of type 2 diabetes is alarmingly high, affecting approximately 300,000 individuals [9]. Of these, approximately a third have established or high-risk CVRD. Notably, the burden of both type 2 diabetes and CVRD in New Zealand is carried more by people of Māori and Pacific descent than other ethnic groups [10, 11]. Māori (the Indigenous people of New Zealand) and Pacific people are more likely to develop type 2 diabetes at younger ages and experience higher rates of complications, including cardiovascular and renal diseases, leading to a reduced life expectancy, than other ethnic groups [12]. These inequities in outcomes are further exacerbated by barriers to accessing effective healthcare [13], with studies reporting that Māori, in general, are less likely to receive adequate medication cover than other ethnic groups in New Zealand [14], although one study reports that this is likely to be a result of clinical inertia and under-prescribing, with Māori and non-Māori equally likely to have medications dispensed once they are prescribed [15].

Despite the widespread availability of SGLT2i internationally for more than a decade, these drugs have only been funded for use in New Zealand since February 2021 [16]. With equitable access to these medications being highlighted as a critical concern, funded access to SGLT2i (empagliflozin) was provided under Special Authority criteria (SAC), with prioritised access for Māori and Pacific people, thereby improving access for groups facing inequitable risk [16]. Initial studies suggest that these SAC have worked as intended, with more than 50% of Māori and Pacific people with type 2 diabetes accessing these drugs in the first 1–3 years, compared with 30–40% of New Zealanders of Asian and European ethnicity [10, 17].

Importantly, while SGLT2i are well documented at decreasing all-cause and CVD-related mortality [18, 19], their effects on mortality in people of different ethnic origins is largely unexplored. Limited studies have reported on SGLT2i use in racially/ethnically diverse groups [20–22], with effects reported as comparable across groups and/or restricted to specific ethnic groups (Black African American, Asian and White) living in distinct geographical regions. Only one study from Australia indicates that SGLT2i can effectively improve clinical measures associated with type 2 diabetes in Indigenous peoples [23], although the effects of these drugs on mortality in these groups have not been evaluated; in addition, the effects have also not been compared with non-Indigenous groups (primarily due to a lack of coded ethnicity data). To date, New Zealand is relatively unique in its ability to systematically record ethnicity in healthcare records, and we have previously reported on how diabetes outcomes, including clinical management and medication use, can differ across different ethnic groups living in the same country [10, 15, 24, 25]. Furthermore, evidence suggests that the efficacy of SGLT2i may vary between population groups [26], but the impact on mortality in Māori and Pacific people is not known. Thus, this study uses a propensity-matched cohort to evaluate and compare mortality outcomes in a primary care population of Māori, Pacific, European (White) and Asian people with type 2 diabetes who did/did not initiate SGLT2i following the funded availability of empagliflozin in February 2021.

## Methods

### Study design

This substudy was part of a larger project assessing the impact of health system factors on inequity in prescribing for type 2 diabetes in primary care in New Zealand, with a particular focus on the newly funded availability of SGLT2i and glucagon-like peptide-1 receptor agonist (GLP-1RA) medications. Ethics approval was provided by the New Zealand Health and Disability Ethics Committee (reference no. 19/CEN/8). This study was reported in accordance with the CONSIDER statement [27], used to strengthen the reporting of research involving Indigenous peoples (see electronic supplementary material [ESM] Table 1). In brief, this study was undertaken under the guidance of a Māori advisory group (led by Māori clinicians/researchers) throughout conception, data collection, analysis and dissemination. The New Zealand Health and Disability Ethics Committee also sets out specific expectations regarding the use of health data, Māori consultation, the impact of the study on Māori (risks and benefits) and the relevance of data sovereignty [28].

### Data sources

Primary care data were sourced directly from four large primary healthcare organisations (PHOs) across the Auckland and Waikato regions of New Zealand, covering an enrolled population of approximately 1 million individuals. Data were extracted and provided for people aged 18–75 years with a diagnosis of type 2 diabetes (Read code C10) recorded in their primary care record as of 1 February 2021 (with this date corresponding to the funded availability of empagliflozin). Extracted individual-level data included National Health Index (NHI; a national identifier that identifies individuals), ethnicity, self-reported gender (male, female or other), age (on 1 February 2021), cardiovascular risk assessment score (CVRA; per cent) and deprivation quintile (NZDep18; provided by the Ministry of Health, where 1 is the lowest decile of socioeconomic deprivation and 10 is the highest), as well as all laboratory results (from 1 August 2019 to 31 December 2023). Ethnicity was coded as recorded in the primary care dataset, according to level 1 classifications of ethnicity as coded by the New Zealand Ministry of Health: this includes European (including New Zealand European, Australian, British, Dutch, Greek, Polish, German, Italian, South Slavic and other European), Māori, Pacific (Samoan, Cook Islands, Tongan, Niuean, Tokelauan, Fijian and other Pacific), Asian (Filipino, Cambodian, Vietnamese, Chinese, Indian, Sri Lankan, Japanese, Korean and other Asian), Middle Eastern, Latin American and African (MELAA) and ‘others’ (all other groups) [29]. In New Zealand, all ethnicity data are self-reported and individuals can identify with multiple ethnic groups. To ensure Indigenous group prioritisation, ethnicity data are coded as Māori (first) and Pacific (second) when multiple ethnicities are reported (each individual was coded with only one ethnicity for analysis) [30]. The initial dataset comprised 59,505 individuals with type 2 diabetes across 302 general practice clinics. This included 12,189 Māori, 10,706 Pacific, 11,040 Asian, 24,145 European, 942 MELAA and 483 ‘others’. New Zealand has a population of approximately 5.5 million so this is about 20% coverage with our dataset. The study regions (Waikato/Auckland) also cover approximately 50% of all Māori and Pacific peoples living in New Zealand and includes large areas of rural and socially deprived communities.

Individuals were deemed eligible for the SGLT2i (empagliflozin) if they were clinically indicated according to the SAC [16] on 1 February 2021. Funded access requires a diagnosis of type 2 diabetes, an HbA_1c_ >53 mmol/mol (7%) despite regular use of at least one glucose-lowering therapy for at least 3 months and any of the following: (1) Māori or Pacific ethnicity; (2) pre-existing CVD (defined as a prior CVD event, congestive heart failure or familial hypercholesterolaemia); (3) a 5-year CVD disease risk of ≥15%; (4) renal disease (an estimated glomerular filtration rate [eGFR] of <60 ml/min per 1.73 m^2^ and/or a urinary albumin/creatine ratio [UACR] of ≥3 mg/mmol in two out of three samples); or (5) a high lifetime risk of cardiovascular or renal disease due to an early diagnosis (taken to be <25 years).

The final study population was formed using the initial population and removing the following: (1) all individuals who identified as MELAA (*n*=942) or ‘other’ ethnicity (*n*=483), who were removed due to small numbers; (2) individuals who were prescribed a GLP-1RA medication (*n*=3249; in New Zealand, individuals meeting the SAC may receive funded access to either SGLT2i or GLP-1RA, but cannot receive both funded concurrently); and (3) individuals without an HbA_1c_ reading in the study period (*n*=9630; required for propensity matching) and those with an eGFR of <30 ml/min per 1.73 m^2^ (*n*=206; indicative of end-stage renal disease). The final study population comprised 45,856 individuals (note that some of the above groups overlap) and details for the population can be found in ESM Table 1. Individuals in the final study population were then grouped into those with CVRD (high-risk and/or established CVD or renal disease, as per the definitions above), those without CVRD and those with unknown CVRD status.

Medication data were sourced from the National Pharmaceutical Collection, a database of all medication-dispensing events nationwide. Drug information was collected for all dispensing events of empagliflozin, metformin, GLP-1RA, insulin and other glucose-lowering therapies for 1 February 2021 to 31 December 2024. The data recorded include the date of dispensing, the formula and the quantity dispensed. Mortality data were sourced from the National Mortality Collection for 1 February 2021 to 31 December 2024 and included the date but not the cause of death.

Individual-level data for two PHOs (PHO1 and PHO2) were available from February 2021 to December 2023, directly aligning with the funded availability of SGLT2i from February 2021. The provision of data from the other two PHOs aligned with the date of data extraction (PHO3 in June 2022 [*n*=4766] and PHO4 in July 2022 [*n*=28,376]) rather than the start of the study period (February 2021). People were included in the study dataset from their date of data availability (February 2021 [PHO1 and PHO2], June 2022 [PHO3] and July 2022 [PHO4]) through to December 2024 (‘the study period’).

### Statistical analysis

To avoid confounding effects of baseline comorbidities and medication use, propensity score matching was performed. Propensity score matching was done in the statistical package R (version 4.4.3; available from https://www.r-project.org/) using the MatchIt package. The nearest neighbour method for 1:1 matching with logistic regression was used, whereby, for each individual, the response variable is whether they initiated empagliflozin during the study period, with covariates based on age, gender, ethnicity, CVRD status (yes/no), baseline HbA_1c_ and use of other diabetes medicines (metformin or insulin), and exact matching for ethnicity was used. Matching was undertaken within PHO groups due to differing observation periods. As the SAC for empagliflozin include an HbA_1c_ threshold for funding eligibility (≥53 mmol/mol [7%]), HbA_1c_ in the matching algorithm required a strict caliper of 0.10 to maintain balance. A standardised mean difference of less than 0.10 was deemed to indicate a negligible difference in means [31].

Values for HbA_1c_, eGFR, body mass index (BMI), CVRA score, LDL-cholesterol, blood pressure (BP) (systolic and diastolic) and UACR were the latest reading in the 18 months preceding the start of the study period. The majority of variables had large proportions of missing values: 16.18% missing for HbA_1c_, 27.9% missing for eGFR, 54.6% missing for BMI, 43.7% missing for CVRA score, 38.4% missing for LDL-cholesterol, 62.2% missing for systolic/diastolic BP and 31.6% missing for UACR. Since propensity matching does not allow for missing data, based upon having the most clinical importance and least missing values, those with missing HbA_1c_ values were removed, and no other laboratory measures were used in the matching algorithm. Post matching, outcomes were calculated for all relevant individuals (e.g. for mean BMI, LDL-cholesterol, etc.), using available data only. No imputation of data was used.

Crude mortality is reported as annualised rates per 1000 people and grouped by ethnicity, CVRD status and SGLT2i use.

To model the effects of empagliflozin on all-cause mortality, Kaplan–Meier curves and Cox proportional hazards models with time-varying covariates were used in R using the survival package on the propensity-matched data. The time-varying aspect of the model allowed for the fact that, while empagliflozin was available from February 2021, the start date of the treatment varied between individuals. To take this into account and to not introduce any survivorship biases into the model, we set the treatment as a time-varying covariate using the approach of Therneau and Grambsch (2000) as reported previously [32]. The final Cox model included age group (<40, 40–49, 50–59, 60–69 and 70+ years), CVRD status, SGLT2i use (yes/no), ethnicity (Māori, Pacific or European), baseline HbA_1c_ group (<50, 50–64, 65–79, 80–94, 95+ mmol/mol) and gender as additive effects and an interaction variable for SGLT2i with ethnicity. The final model satisfied the proportional hazards assumption with a global *p* value of 0.136 when using the cox.zph function from the survival package. Note that the Cox model analysis included only those individuals who identified as European, Māori or Pacific, as the Asian ethnicity group was removed because that group violated the proportional hazards assumption.

### Role of the funding source

The Health Research Council of New Zealand funded this study but played no role in the study design or in the collection, analysis and interpretation of data; in the writing of the report; or in the decision to submit the paper for publication.

### Ethics approval and consent to participate

This study was approved by the Health and Disability Ethics Committee of New Zealand (ref: 19/CEN/8) and conforms to the Declaration of Helsinki. Consent for publication is not applicable as only deidentified data were analysed.

## Results

### Annualised crude mortality

The annualised crude all-cause mortality rates for all individuals with/without CVRD who did/did not undertake therapy with the SGLT2i are given in Table 1. Māori had the highest mortality rate, both with (55.81 deaths per 1000 individuals per year) and without CVRD (14.15 deaths per 1000 individuals per year), with rates nearly twice as high as those of European and Pacific descent, and three or four times higher than Asian people (Table 1). Among those with CVRD, compared with non-users of SGLT2i, those receiving SGLT2i medications had lower mortality rates in all ethnic groups, although the improvement was highest for Māori and Pacific people (rate ratios of 0.29 and 0.32, respectively; all *p*≤0.002; Table 1). For those without CVRD, SGLT2i use significantly reduced the annualised crude mortality rate in all ethnic groups except for Asian people (Table 1).

**Table 1:** Crude annualised all-cause mortality rate (per 1000 people) for individuals with type 2 diabetes with and without CVRD who did/did not initiate SGLT2i use in the study period following funded availability in February 2021

	CVRD					Non-CVRD				
	All	Māori	European	Asian	Pacific	All	Māori	European	Asian	Pacific
Non-SGLT2i users (*N*=27,185)										
*n*	11,822	2483	5404	2074	1861	15,363	1937	7987	3888	1551
Deceased, *n*	1114	386	490	83	155	315	76	175	35	29
Deaths per 1000 people per year	35.24	55.81	33.09	15.79	33.49	7.69	14.15	8.03	3.53	7.51
SGLT2i users (*N*=18,671)										
*n*	11,556	2842	3764	1781	3169	7115	1411	2414	1416	1874
Deceased, *n*	396	126	148	39	83	67	20	27	7	13
Deaths per 1000 people per year	13.09	16.24	14.52	8.71	10.62	3.62	5.20	4.16	1.97	2.81
Rate ratio	0.37	0.29	0.44	0.55	0.32	0.47	0.37	0.52	0.56	0.37
95% CI	0.33, 0.42	0.24, 0.36	0.36, 0.53	0.37, 0.82	0.24, 0.42	0.36, 0.61	0.21, 0.61	0.33, 0.78	0.21, 1.27	0.18, 0.74
*p* value	<0.001	<0.001	<0.001	0.002	<0.001	<0.001	<0.001	<0.001	0.20	0.004

### Survival rate modelling following propensity matching

Following propensity matching, two groups (with/without initiation of SGLT2i), each containing 12,792 individuals, were included for analysis (with 2604 Māori, 5214 European, 2537 Asian and 2437 Pacific people in each group). Post matching, all variables were checked, and no major standardised mean differences (>0.1) were found between groups other than for BMI (Table 2). The clinical and demographic characteristics of these groups are given in Table 2, while the characteristics of the SGLT2i group alongside the entire type 2 diabetes study population are given in ESM Table 2.

**Table 2:** Baseline clinical and demographic characteristics of the final propensity-matched population

Characteristic	Propensity-matched SGLT2i non-users					Propensity-matched SGLT2i users					SMD for all
	Māori (*n*=2604)	European (*n*=5214)	Asian (*n*=2537)	Pacific (*n*=2437)	All (*n*=12,792)	Māori (*n*=2604)	European (*n*=5214)	Asian (*n*=2537)	Pacific (*n*=2437)	All (*n*=12,792)	
Age, years	56.67 (13.75)	60.82 (12.42)	57.47 (11.55)	56.39 (12.69)	58.47 (12.75)	55.89 (10.89)	61.31 (9.69)	56.21 (11.23)	54.92 (10.66)	57.98 (10.81)	0.041
Gender, % male	50.69	63.64	60.94	46.74	57.25	47.85	64.21	59.01	44.36	56.07	0.027
Social deprivation quintile	4.01 (1.22)	3.01 (1.38)	3.24 (1.35)	4.18 (1.09)	3.48 (1.38)	4.03 (1.17)	3.10 (1.36)	3.27 (1.40)	4.22 (1.08)	3.54 (1.37)	0.038
Urban, % yes	72.40	78.77	97.19	96.86	84.49	74.91	77.67	97.16	97.02	84.40	0.003
Baseline HbA_1c_, mmol/mol,	63.45 (20.87)	62.49 (16.64)	60.31 (14.27)	62.24 (18.41)	62.21 (17.54)	66.56 (19.58)	62.64 (14.84)	61.93 (13.55)	65.39 (17.98)	63.82 (16.40)	0.095
Baseline HbA_1c_, %	7.96 (1.91)	7.87 (1.52)	7.67 (1.31)	7.85 (1.68)	7.84 (1.60)	8.24 (1.79)	7.88 (1.36)	7.82 (1.24)	8.13 (1.64)	7.99 (1.50)	0.095
BMI, kg/m^2a^	35.77 (8.98)	32.44 (7.16)	27.66 (5.56)	35.64 (8.87)	32.75 (8.25)	37.43 (8.67)	34.61 (7.75)	28.56 (6.59)	37.41 (8.95)	34.43 (8.68)	0.199
CVRA score, median, (IQR)^a^	12 (6,18)	10 (6,16)	6 (4,11)	7 (4,11)	9 (5,15)	10 (6,17)	11 (7,17)	6 (4,10)	7 (4,11)	9 (5,15)	0.027
LDL-cholesterol^a^	2.42 (1.04)	2.34 (0.97)	2.23 (0.91)	2.26 (0.98)	2.31 (0.98)	2.39 (0.96)	2.26 (0.95)	2.11 (0.89)	2.20 (0.94)	2.24 (0.94)	0.077
Systolic BP, mmHg	131.10 (19.99)	131.91 (17.63)	130.56 (16.54)	131.58 (18.51)	131.51 (18.35)	131.20 (19.44)	133.21 (17.10)	131.28 (17.29)	131.57 (17.63)	132.31 (17.92)	0.044
Diastolic BP, mmHg^a^	80.24 (14.69)	77.96 (11.41)	78.30 (10.23)	80.82 (14.02)	78.86 (12.62)	81.63 (14.93)	78.61 (12.12)	78.61 (10.53)	79.80 (11.56)	79.61 (12.96)	0.059
CVRD, % yes	66.74	55.91	49.82	62.21	58.11	59.10	57.59	50.65	52.48	55.55	0.052
eGFR, ml/min per 1.73 m^2^^a^	76.48 (18.58)	78.08 (15.96)	82.04 (14.22)	76.02 (18.30)	78.18 (16.76)	80.58 (14.50)	78.63 (15.05)	82.32 (13.23)	78.57 (15.72)	79.73 (14.80)	0.098
log(UACR)^a^	1.49 (1.82)	0.69 (1.36)	0.89 (1.32)	1.44 (1.73)	1.03 (1.57)	1.36 (1.68)	0.85 (1.40)	1.09 (1.44)	1.33 (1.56)	1.10 (1.51)	0.041
Dispensed medications^b^											
Insulin, % yes	25.31	32.87	16.59	20.07	25.66	26.65	31.89	23.33	23.02	27.44	0.04
Metformin, % yes	79.95	82.80	91.76	84.94	84.40	79.92	85.29	89.32	85.02	84.94	0.015
Statins, % yes	73.35	77.23	77.02	72.26	74.45	82.45	85.62	84.59	78.95	83.50	0.200
ACEis/ARBs, % yes	73.58	71.86	68.86	75.7	72.35	82.03	81.09	77.26	80.88	80.48	0.192

Data are presented as mean (SD) unless otherwise stated

^a^ Missing data for these measures are as follows (SGLT2i non-use/use): BMI (47.8%/44.0%), CVRA score (35.7%/30.1%), LDL-cholesterol (14.5%/13.8%), BP (39.1%/39.2%), eGFR (15.4%/14.5%), UACR (16.8%/11.9%)

^b^ Medications were coded as ‘yes’ if individuals were prescribed these agents at least once during the study period

ACEi, angiotensin-converting enzyme inhibitor; ARB, angiotensin receptor blocker; SMD, standardised mean difference

**Fig. 1:**
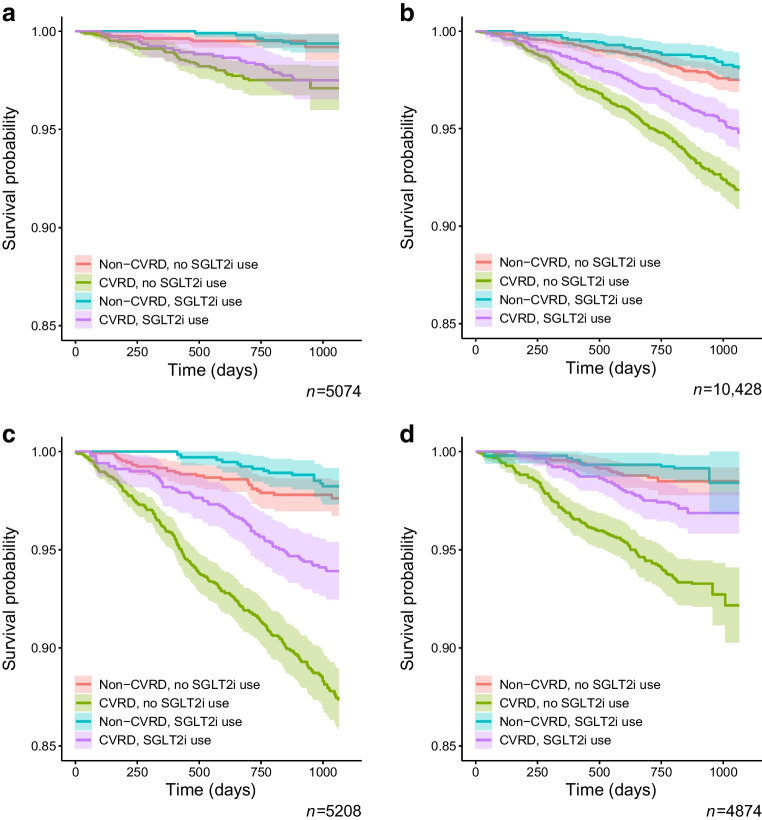
Kaplan–Meier survival curves for (**a**) Asian, (**b**) European, (**c**) Māori and (**d**) Pacific people with type 2 diabetes (with/without CVRD) and use/no use of SGLT2i

The use of SGLT2i was associated with a lower likelihood of mortality for Māori, Pacific and European people, but not Asian people, with type 2 diabetes. After adjusting for ethnicity, CVRD status, age group, baseline HbA_1c_ and gender, Māori exhibited the greatest reduction in mortality (HR 0.475; 95% CI 0.336, 0.672; *p*<0.001), followed by Pacific (HR 0.507; 95% CI 0.395, 0.651; *p*<0.001) and European people (HR 0.667; 95% CI 0.545, 0.816; *p*<0.001). The Kaplan–Meier plots are given in Fig. 1. There was no significant interaction between CVRD status and SGLT2i use in the model (*p*>0.05).

For those not using SGLT2i, comparing Māori to European people, the Cox model HR was 1.96 (95% CI 1.66, 2.30; *p*<0.001), which decreased to 1.48 (95% CI 1.12, 1.96; *p*=0.005) for those using SGLT2i. For Pacific vs European people, the HR was significantly different in those not using the SGLT2i (HR 1.34; 95% CI 1.09, 1.64; *p*=0.004) but was comparable in those using the medication (HR 0.96; 95% CI 0.67, 1.36; *p*=0.80). Māori continued to have a higher likelihood of mortality than Pacific people, both without (HR 1.46; 95% CI 1.19, 1.79; *p*<0.001) and with (HR 1.55; 95% CI 1.07, 2.26; *p*=0.020) SGLT2i medication use.

The impact of other variables on mortality is provided in ESM Table 3. In brief, mortality was more likely in people of Māori or Pacific ethnicity (vs European), in those with (vs those without) CVRD, in men (vs women) and in those with advancing age and higher HbA_1c_ levels.

## Discussion

While the effects of SGLT2i use on health outcomes, including mortality, have been described previously [1–3, 33, 34], our study is one of the first to characterise these effects in people of different ethnicities in a multicultural population. Importantly, we note that the size of the effect of SGLT2i use on mortality appears to be ethnicity dependent, with the greatest improvements observed among Māori and Pacific people. While our study, in general, agrees that the use of SGLT2i in type 2 diabetes decreases mortality rates [18, 34], it differs from the limited published literature that reports that SGLT2i use shows consistent efficacy on cardiovascular death across different ethnic groups, notably Asian, White and Black individuals [20]. However, we note the lack of more nuanced ethnicity identification data in this meta-analysis study [20].

Furthermore, while there are limited studies reporting on the efficacy of SGLT2i for improving outcomes in Indigenous and minority populations [23, 35, 36], there seems to be an almost complete lack of data reporting on mortality outcomes of SGLT2i use between ethnic groups at a more granular level. This largely reflects the lack of available data on race and ethnicity in many studies [37, 38]. Ethnicity is coded at four sublevels in New Zealand data [29], although we note that few countries record ethnicity at this level in health data. Other countries collect ethnicity data in census or hospital data only [39], and we suggest that there are opportunities via prospective data collection and/or data linkage to census collections to better understand the effects of medications on people from specific ethnic groups, particularly as race, ethnicity and ancestry are increasingly common classification variables used in health research [40, 41].

In addition, there appear to be no data on mortality outcomes with SGLT2i use in Indigenous people vs their non-Indigenous counterparts. Data are urgently required to address these gaps, as countries such as Australia and Canada, as well as New Zealand, continue to report on persistent inequity of outcomes for these population groups. In addition, studies report no difference in glycaemic outcomes between Asian and non-Asian individuals using SGLT2i [22], although our data suggest that this may not translate into comparable mortality outcomes.

While our study supports the use of SGLT2i across all ethnic groups, our results underscore the importance of these medications for Māori and Pacific people, as evidenced by significantly greater survival improvements with SGLT2i use compared with other groups. The causes are likely multifactorial, although in New Zealand there are long-standing inequities around healthcare access [13], particularly for Māori and Pacific people. Furthermore, while we did not have data on the duration of disease, it is well established that Māori and Pacific people with type 2 diabetes are often diagnosed at an earlier age than New Zealand European counterparts and have earlier development of complications alongside lower access to medications [14]. It is plausible that the Māori and Pacific individuals in our study had more advanced disease, which correlates with higher baseline HbA_1c_ values and the use of statins and antihypertensive medications (Table 2). These groups also associate with higher baseline BMI values, although studies suggest that weight loss with SGLT2i medications is comparable across BMI categories [42]. Furthermore, despite the fact that Indigenous populations often experience higher background cardiometabolic and kidney risk [43, 44], there is also limited evidence that Indigenous groups may have specific renin/angiotensin/aldosterone system (RAAS) genetic markers [45] that could affect their susceptibility to chronic CVRD. This has not been explored in the context of our New Zealand cohort, but it is possible that ethnicity and genetics may alter the efficacy of drugs that impact the RAAS, such as SGLT2i. Further studies are required to elucidate possible associations between ethnicity and drug efficacy.

Funded access to SGLT2i in New Zealand is via SAC, with prioritised access for Māori and Pacific people [16]. Although contentious at the time, this was intended to reduce the inequities in health outcomes for these groups with type 2 diabetes [17]. However, data recently published by our team show that this method of ethnic prioritisation has resulted in significantly higher uptake of these medications among Māori and Pacific people than among other ethnic groups in New Zealand [10, 17], with current, unpublished Procare PHO data from A. Moffitt showing that 75% of all eligible Māori and Pacific people had initiated SGLT2i by the end of 2024. This is a dramatic result when compared with other countries that report rates of SGLT2i use of 8.7–16.1% (e.g. the DISCOVER study [46]). The DISCOVER study also suggests that the highest prevalence of SGLT2i use was by cardiologists (26.1%), suggesting that this may also include treatment for heart failure, something that was not funded or approved for use in New Zealand at the time of our study.

Importantly, we do note that there are limitations with our study. Propensity matching was a key factor of our study design, yet it relies on appropriately balancing baseline data. In our dataset, there was a high level of missing data for key clinical measures such as renal function, BP lipids and other medications, which restricted the matching of covariates and potential selection bias. We also note that there were limited data for people with particularly high HbA_1c_ (>100 mmol/mol [11.3%]), with unmatched people removed from the analysis population. Second, we note that Māori initiating SGLT2i had a slightly higher mean baseline HbA_1c_ than non-users (66.6 vs 63.5 mmol/mol [8.25% vs 8.0%]), which may have contributed to the results. Third, we acknowledge that other medications such as dipeptidyl peptidase 4 (DPP4) inhibitors and sulfonylureas may have influenced these findings, but these were not included in the propensity matching. These were not included due to smaller numbers, although a meta-analysis of randomised controlled trials suggested that there is a neutral effect of DPP4 and SGLT2i. Similarly, we did not have data on coronary artery disease or heart failure, both of which are conditions that may have impacted these study findings. Fourth, we acknowledge that individuals prescribed with SGLT2i may differ systematically from non-users in ways not fully captured in the dataset. SGLT2i use in our study may be associated, for example, with higher healthcare engagement, consistent medication possession, higher baseline medication use, less frailty and other things that promote lower mortality risk. Fifth, our data rely on ethnicity, as they are coded in the New Zealand health data: while this is self-reported by individuals, we do acknowledge that this limits the analytic granularity of our study. We also note that stratification was done across the entire dataset, although individual clinics may have large variations in delivery of care. These variables should be explored in further studies, although matching and stratification within clinics is generally limited by sample size. Regardless, our data do provide real-world outcome data for this population.

Finally, our study reports on all-cause mortality, yet SGLT2i drugs are most effective for improving cardiovascular-related outcomes. Coded mortality data were not available for this cohort, although the data suggest that up to a third of people with type 2 diabetes may now die of cancer, with decreases being seen in cardiovascular deaths overall [47]. As such, we suggest that further studies should explore the effects of SGLT2i use specifically in CVD deaths in people of differing ethnicities, particularly as it has been suggested that SGLT2i use can reduce the risk of CVD death by up to 32% [33].

### Conclusions

SGLT2i medications offer significant potential to reduce mortality in individuals with type 2 diabetes, although the greatest real-world benefit seen in our study was in Indigenous Māori and Pacific people. While the reasons for this are multifactorial, these findings do support prioritised access of these medications for these populations, providing a valuable tool for improving health equity. Further international studies should collect ethnicity data wherever possible such that the impact of these medications on other ethnic groups (including Indigenous and minority groups) can be more comprehensively explored.

## Supplementary Information

Below is the link to the electronic supplementary material.

ESM Tables (PDF 233 KB)

## Data Availability

The data that support the findings of this study are available from the corresponding author but restrictions apply to the availability of these data due to patient privacy. These data were supplied and used under licence for the current study and therefore are not publicly available. Data are, however, available from the authors upon reasonable request and with the permission of the Procare, Pinnacle and Hauraki PHOs and the National Hauora Coalition.

## Abbreviations

BMI: Body mass index
BP: Blood pressure
CVD: Cardiovascular disease
CVRA: Cardiovascular risk assessment score
CVRD: Cardiovascular and/or renal disease/risk
DPP4: Dipeptidyl peptidase 4
eGFR: Estimated glomerular filtration rate
GLP-1RA: Glucagon-like peptide-1 receptor agonist
MELAA: Middle Eastern, Latin American and African
NHI: National Health Index
PHO: Primary healthcare organisation
RAAS: Renin/angiotensin/aldosterone system
SAC: Special Authority criteria
SGLT2i: Sodium-glucose cotransporter 2 inhibitor(s)
UACR: Urinary albumin/creatine ratio
