# Correction: SGLT2 inhibitor use and disparities in all-cause mortality in type 2 diabetes: insights from a multi-ethnic population

**DOI:** 10.1007/s00125-026-06752-z

**Published:** 2026-05-22

**Authors:** Lynne Chepulis, Han Gan, David Simmons, Mark Rodrigues, Rawiri Keenan, Rinki Murphy, Tim Kenealy, Leanne Te Karu, Dianna Magliano, Jo Scott-Jones, Allan Moffitt, Chunhuan Lao, Ross Lawrenson, Ryan G. Paul

**Affiliations:** 1 Medical Research Centre, University of Waikato, Hamilton, New Zealand; 2 School of Computing and Mathematical Sciences, University of Waikato, Hamilton, New Zealand; 3 School of Medicine, Western Sydney University, Sydney, NSW Australia; 4 Faculty of Medical and Health Sciences, University of Auckland, Auckland, New Zealand; 5 Health New Zealand / Te Whatu Ora Te Toka Tumai, Auckland, New Zealand; 6 School of Public Health and Preventative Medicine, Monash University, Melbourne, VIC Australia; 7 Midlands Health Network, Hamilton, New Zealand; 8 Procare Health Limited, Auckland, New Zealand; 9 Health New Zealand / Te Whatu Ora Waikato, Hamilton, New Zealand

**Correction: Diabetologia**

10.1007/s00125-026-06733-2

In the original version of the article, the keys in Fig. 1 were incorrect. Following is the corrected figure:

**Fig. 1:**
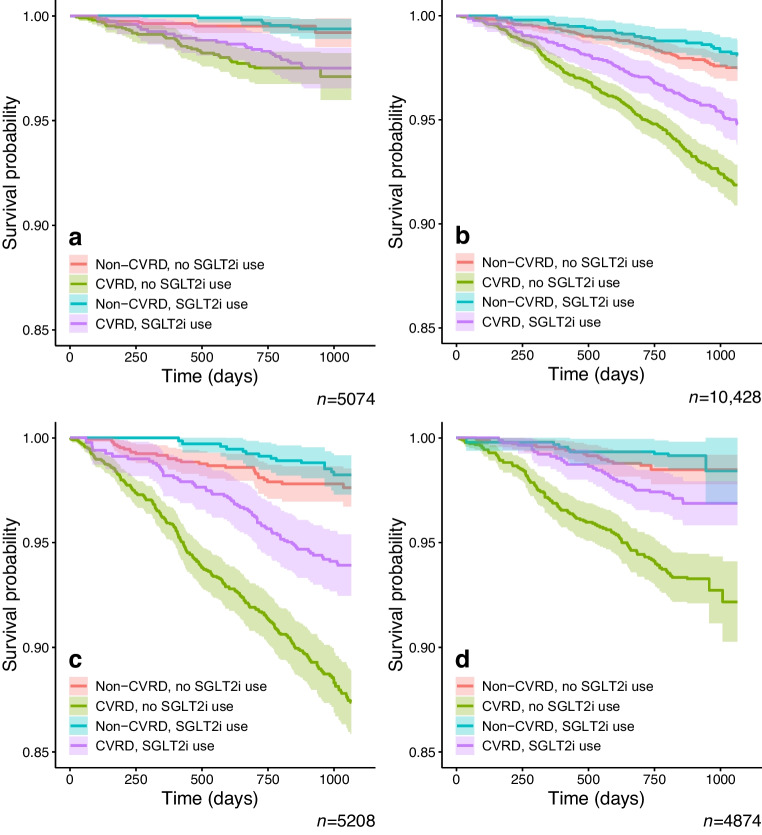
Kaplan–Meier survival curves for (**a**) Asian, (**b**) European, (**c**) Māori and (**d**) Pacific people with type 2 diabetes (with/without CVRD) and use/no use of SGLT2i

The original article has been corrected.

