# Supplementary material for: SGLT2 inhibitor use and disparities in all-cause mortality in type 2 diabetes: insights from a multi-ethnic population
Source: Diabetologia. 2026 Apr 27;69(8):2189–99. doi: 10.1007/s00125-026-06733-2 (PMC13310227; doi:10.1007/s00125-026-06733-2)
Supplement: Supplementary file 1 — ESM Tables (PDF 233 KB) [file 125_2026_6733_MOESM1_ESM.pdf]

Electronic supplementary Table 1 Inclusion / Response to the consolidated criteria for strengthening reporting of health research involving indigenous peoples: the CONSIDER statement

**Item Checklist Item**		
Governance		Response / Inclusion in Manuscript
**1.**	Describe partnership agreements between the research institution and Indigenous-governing organization for the research, (e.g., Informal agreements through to MOU (Memorandum of Understanding) or MOA (Memorandum of Agreement)).	While the project has a focus on Indigenous group outcomes, and this research was not directly dealing with patients and community (i.e. only data), more specific agreements were not sought.<br>All data were sourced directly from Primary Healthcare Organisations (PHOs) across the region. Two of these are Indigenous led and governed, with the others being more 'mainstream' though still having their own requirements and processes for Māori consultation and engagement protections.<br>All aspects of the project required ethics approval and locality / data governance approvals for each of these organisations and the respective geographical locations they serve (both ethics and locality/ data governance require Māori consultation as a part of the process).<br><br>The University of Waikato as a whole acknowledges and recognises Waikato Tainui as Mana Whenua, the people of the land the University stands on. This is both through formal agreements as owners of the land the university is situated on, as well as part of Iwi/Indigenous governance of the university overall.
**2.**	Describe accountability and review mechanisms within the partnership agreement that addresses harm minimization.	Harm is minimalized by ensuring that reporting is strengths based and that research is undertaken using culturally safe practices. Part of this is our strong representation of Indigenous named researchers as part of the team undertaking this project. Analysis and publications are undertaken with these senior Māori researchers to ensure the framing is undertaken in a way to minimise harm.
**3.**	Specify how the research partnership agreement includes protection of Indigenous intellectual property and knowledge arising from the research, including financial and intellectual benefits generated (e.g., development of traditional medicines for	As a retrospective review of data there is minimal opportunity for IP and commercial development.

	commercial purposes or supporting the Indigenous community to develop commercialization proposals generated from the research).	
Prioritization		
**4.**	Explain how the research aims emerged from priorities identified by either Indigenous stakeholders, governing bodies, funders, non-government organization(s), stakeholders, consumers, and empirical evidence	This research is grounded in well-established Māori health priorities, particularly the disproportionate burden of type 2 diabetes and higher rates of complications in Māori compared to other ethnic groups. These priorities have been repeatedly articulated by Māori health leaders, iwi and Māori health providers, and national Māori health strategies. This wider study was funded due to inequities in diabetes management being a funder priority.
Relationships (Indigenous stakeholders/participants and Research team)		
**5.**	Specify measures that adhere and honor Indigenous ethical guidelines, processes, and approvals for all relevant Indigenous stakeholders, recognizing that multiple Indigenous partners may be involved, e.g., Indigenous ethics committee approval, regional/national ethics approval processes.	We sought review and endorsement from relevant Māori or Indigenous stakeholders and ethics committees, such as iwi- or regionally based Māori Research Review Committees and Māori Research Advisory Groups. Our research is explicitly aligned with Te Ara Tika (Māori research ethics) following the principles of Te Mana Raraunga for data governance. Māori consultation is required for both ethics and PHO locality approval to access data and/or to undertake research.
**6.**	Report how Indigenous stakeholders were involved in the research processes (i.e., research design, funding, implementation, analysis, dissemination/recruitment).	Multiple Māori staff are named investigators on this wider programme of work and this specific piece of work. The involvement of senior Māori researchers Dr Rawiri Keenan, Dr Leanne te Karu (Muaūpoko; Te Ati Haunui-a-Pāpārangi), Dr Ryan Paul, (Ngāti Maru) have guided all aspects of the research conception, design, analysis, interpretation and dissemination.
**7.**	Describe the expertise of the research team in Indigenous health and research	Dr Rawiri Keenan is a Māori GP with extensive research and leadership experience across primary care. Dr Leanne Te Karu is a Māori pharmacist prescriber who is involved nationally and regionally in many leadership roles in the health system. Dr Ryan Paul is a diabetes specialist and endocrinologist. He is also co-chair of Mahitahi Matehuka (National Diabetes Network) and Clinical Director of the Waikato Diabetes Regional Diabetes Service. All three Māori staff have significant combined experience across Indigenous health and research.
Methodologies		
**8.**	Describe the methodological approach of the research including a rationale of methods used and implication for Indigenous stakeholders, e.g., privacy and confidentiality (individual and collective)	This is a retrospective review of existing datasets. This research uses an applied, health equity-oriented approach to generate evidence with direct relevance to support the use of SGLT2i medications for Māori

		peoples. All data were deidentified and only reported as aggregated data. The existing datasets as well as the new dataset created from this study are all encrypted, stored securely and uses unique identifiers to ensure confidentially.
**9.**	Describe how the research methodology incorporated consideration of the physical, social, economic and cultural environment of the participants and prospective participants. (e.g., impacts of colonization, racism, and social justice). As well as Indigenous worldviews.	Our research team recognise the significance of ongoing colonialism and the impact that this has on health outcomes. Our methodology acknowledges that the health data used in this study is largely collected from primary care clinics oriented to a Westernised framework and that this may influence the results observed.
Participation		
**10.**	Specify how individual and collective consent was sought to conduct future analysis on collected samples and data (e.g., additional secondary analyses; third-parties accessing samples (genetic, tissue, blood) for further analyses).	Collective consent was obtained at clinic level, with an opt-in process within each Primary Healthcare Organisation. Individual patient is obtained at the time that a patient joins a particular practice (they can opt in / our of their data be utilised for research purposes). No further analyses have been undertaken, nor third party access.
**11.**	Described how the resource demands (current and future) placed on Indigenous participants and communities involved in the research were identified and agreed upon including any resourcing for participation, knowledge, and expertise	This was a retrospective review of health data only, so there were / are no demands on any individual participants.
**12.**	Specify how biological tissue and other samples including data were stored, explaining the processes of removal from traditional lands, if done, and of disposal.	This was a retrospective review of health data only.
Capacity		
**13.**	Explain how the research supported the development and maintenance of Indigenous research capacity (e.g., specific funding of Indigenous researchers).	The majority of the work for the project was undertaken by the named investigators included as authors including the aforementioned senior Māori academics and clinicians. Whilst we hoped to support an Indigenous researcher for the statistical analysis undertaken in this specific study, we were unable to recruit one for this project.<br><br>We have, however, supported and employed a number of other Indigenous staff to undertaken other studies within this larger programme of work (e.g qualitative studies).<br><br>As mentioned, two of the PHOs who worked with us for data access on this project are Indigenous led and governed. Their involvement at a PHO and individual clinics within their network (with a high proportion of Indigenous population) have partnered with us on subsequent projects. This has included (Māori) clinical and non-clinical

		staff being employed as part of ongoing research projects, growing their own portfolios and research capacities.
**14.**	Discuss how the research team undertook professional development opportunities to develop the capacity to partner with Indigenous stakeholders?	As this was a retrospective data review only we have not have any opportunity to build capacity with our Indigenous stakeholders. However, this study is part of a larger program of work across diabetes and cardiovascular disease in primary care, and as such we continue to strength our partnerships with the Indigenous-led PHOs , including working with junior Māori GPs to increase their skills in health research.
Analysis and interpretation		
**15.**	Specify how the research analysis and reporting supported critical inquiry and a strength-based approach that was inclusive of Indigenous values.	This study takes a strength-based approach to understand how prioritised access to specific medications for Māori can potentially lead to significantly improved health outcomes. Our research has been led through a equity-focussed approach.
Dissemination		
**16.**	Describe the dissemination of the research findings to relevant Indigenous governing bodies and peoples.	In addition to the below (17) dissemination to facilitate change within the healthcare system, we will be sharing back to our PHO partners who facilitated data access. This is to ensure their clinicians are aware, but also highlight the power of their data in generating new knowledge. While not explicit partners to the project several groups such as Te Ohu Rata (Māori doctors), and Iwi-Māori partnership boards (Iwi leadership bodies with a focus on health) among others are identified as key audiences to share our published findings with.
**17**	Discuss the process for knowledge translation and implementation to support Indigenous advancement (e.g., research capacity, policy, investment).	Early findings have been shared with national bodies interested in mortality reduction. Following publication several local conferences that are both clinician and community facing are planned for submission. While this is of importance to Māori, much of this will be shared to 'mainstream' channels as that is where the majority of clinicians who can implement changes/prescribe the medications indicated from this research.

Electronic supplementary Table 2: Baseline clinical and demographic characteristics of the initial T2D study population

	Entire T2D study population (n = 45,856)					SGLT2i users (n=18,671)				
	**Māori 8,673**	**European 19,569**	**Asian 9,159**	**Pacific 8,455**	**ALL 45,856**	**Māori 4,253**	**European 6,178**	**Asian 3,197**	**Pacific 5,043**	**ALL 18,671**
**Age (mean ± SD)**	56·15 (12·54)	60·12 (12·46)	56·95 (11·62)	55·35 (11·62)	57·85 (12·33)	55·60 (10·95)	61·26 (9·64)	56·14 (11·20)	54·56 (10·82)	57·28 (10·92)
**Gender (% Male)**	48·92	59·84	56·14	47·34	54·73	52·32	66·49	60·84	49·87	57·81
**Social Deprivation Quintile (mean ± SD)**	4·03 (1·18)	3·01 (1·37)	3·19 (1·38)	4·20 (1·09)	3·46 (1·39)	4·06 (1·16)	3·11 (1·37)	3·30 (1·38)	4·24 (1·07)	3·66 (1·35)
**Urban (% yes)**	74·40	78·46	96·86	97·16	84·65	77·24	78·02	97·17	97·66	86·17
**Baseline HbA1c (mmol/mol, mean ± SD)**	64·55 (21·23)	58·15 (16·11)	58·05 (14·49)	66·63 (19·45)	60·90 (17·92)	72·40 (20·60)	65·41 (16·20)	65·29 (15·30)	72·18 (18·88)	68·81 (18·22)
**BMI (kg/m2, mean ± SD)**	36·77 (9·24)	32·93 (7·93)	27·89 (6·38)	36·53 (8·80)	33·32 (8·75)	37·51 (9·46)	34·63 (8·32)	28·66 (6·75)	37·05 (8·66)	34·89 (8·99)
**CVRA score (median [IQR])**	11 [11]	9 [10]	6 [7]	7 [7]	8 [9]	11 [11]	11 [10·75]	7 [8]	7 [7]	10 [11]
**LDL-Cholesterol (mean ± SD)**	2·45 (1·03)	2·40 (0·99)	2·26 (0·94)	2·25 (0·97)	2·35 (0·99)	2·37 (1·02)	2·24 (0·96)	2·12 (0·91)	2·20 (0·95)	2·23 (0·96)
**Systolic BP**	131·17 (19·58)	132·11 (17·57)	129·58 (17·13)	131·36 (17·88)	131·54 (18·13)	131·35 (19·39)	133·24 (17·25)	131·40 (17·43)	130·67 (17·21)	132·22 (18·08)
**Diastolic BP**	80·92 (14·53)	78·19 (11·73)	77·70 (10·61)	80·03 (12·01)	78·98 (12·54)	81·91 (14·85)	78·67 (12·11)	78·46 (10·62)	79·69 (11·06)	79·88 (13·05)
**CVRD[1] (%Yes)**	61·40	46·85	42·09	59·49	50·98	66·82	60·93	55·71	62·84	61·89
**eGFR (mean ± SD)**	78·77 (16·50)	79·10 (14·99)	82·67 (13·16)	77·56 (16·82)	79·48 (15·37)	80·21 (14·96)	78·54 (15·14)	81·90 (13·76)	78·15 (16·18)	79·40 (15·21)
**log(UACR) (mean ± SD)**	1·44 (1·75)	0·63 (1·36)	0·86 (1·35)	1·48 (1·66)	1·00 (1·54)	1·62 (1·71)	0·94 (1·43)	1·20 (1·48)	1·58 (1·62)	1·32 (1·59)
**Insulin (%Yes)**	26·03	30·51	16·82	24·16	25·76	33·04	34·70	26·84	29·21	31·49
**Metformin (%Yes)**	73·19	64·69	80·47	82·78	72·78	86·57	87·31	91·12	90·86	88·75

[1]Established cardiovascular and/or renal disease and/or elevated risk as per special authority criteria (16, 17)

Electronic supplementary Table 3: Hazard rate ratios for mortality based on other variables

Variable (Reference)	HR	95% CI	p-value
**Ethnicity (European)**			
**Māori**	1·957	(1·664, 2·301)	<0·001
**Pacific**	1·343	(1·098, 1·642)	0·004
**CVRD (No CVRD)**	2·585	(2·172, 3·076)	<0·001
**Age Group (<40); years**			
**40-49**	1·690	(0·975, 2·930)	0·061
**50-59**	2·996	(1·831, 4·901)	<0·001
**60-69**	5·799	(3·587, 9·375)	<0·001
**70+**	8·628	(5·307, 14·028)	<0·001
**HbA1c (<50); mmol/mol**			
**50-64**	0·974	(0·821, 1·157)	0·767
**65-79**	1·444	(1·186, 1·758)	<0·001
**80-94**	1·433	(1·100, 1·868)	0·007
**95+**	2·166	(1·683, 2·787)	<0·001
**Male (Female)**	1·257	(1·106, 1·429)	<0·001
